# A new species of *Rhopalopsole* (Plecoptera, Leuctridae) from Yunnan Province, China

**DOI:** 10.3897/BDJ.12.e134258

**Published:** 2024-10-31

**Authors:** Xiao Yang, Yu-Zhou Du

**Affiliations:** 1 School of Plant Protection & Institute of Applied Entomology, Yangzhou University, Yangzhou 225009, China School of Plant Protection & Institute of Applied Entomology, Yangzhou University Yangzhou 225009 China

**Keywords:** *Rhopalopsole*, Plecoptera, Leuctridae, new species, Yunnan Province, China

## Abstract

**Background:**

A new species of the genus *Rhopalopsole* (Plecoptera, Leuctridae) from Yunnan Province, China, *Rhopalopsoledulongjianga* **sp. nov.**, is described and illustrated.

**New information:**

This new species is compared to the similar species, *Rhopalopsolebispina* (Wu, 1949). Additionally, we provide a supplemental description and colour plates of *R.bispina*. A key to *Rhopalopsole* species from Yunnan Province, China, is also provided in this paper.

## Introduction

*Rhopalopsole* Klapálek, 1912 is a species-rich genus in the family Leuctridae, with more than 80 valid species known from the Oriental and eastern Palaearctic Regions (Klapálek 1912, Okamoto 1922, Wu 1949, Jewett 1958, Illies 1966, Kawai 1967, Kawai 1968, Kawai 1969, Wu 1973, Jewett 1975, Harper 1977, Zwick 1977, Sivec et al. 2008, Stark and Sivec 2008, Harrison and Stark 2008, Stark et al. 2012, Li et al. 2017, DeWalt et al. 2024). Currently, more than 50 species of this genus have been recorded from China, with recent contributions made by Yang et al. (2004), Yang and Li (2006), Sivec et al. (2008), Yang et al. (2009), Li and Yang (2010), Li et al. (2010a), Li et al. (2010b), Li and Yang (2011), Li et al. (2011), Qian and Du (2011), Li and Yang (2012), Qian and Du (2012a), Qian and Du (2012b), Qian and Du (2013), Qian et al. (2014), Chen and Du (2017), Li et al. (2017), Qian and Du (2017), Mo et al. (2018), Yang and Du (2021a), Yang and Du (2021b), Yang and Du (2021c), Yang et al. (2021), Chen et al. (2022), Li et al. (2022), Yang et al. (2022), Yang and Du (2024).

Yunnan Province is located on the south-western border of China, bordering Myanmar, Laos and Vietnam. The northern part of Yunnan Province belongs to the southwest region and the southern part belongs to the South China Region. Yunnan Province belongs to the Oriental realm and has many large rivers flowing through it, with significant differences in elevation. Its unique geographical location and favourable climatic conditions provide an excellent environment for Leuctridae insects. However, identification is challenging due to the simplicity of some species drawings and the lack of colour pictures. Therefore, it is necessary to reorganise and summarise the Leuctridae species of Yunnan Province.

Recently, we visited Yunnan Province to survey aquatic insect diversity and collected and examined a batch of Plecoptera specimens. We describe and illustrate a new species of *Rhopalopsole* Klapálek, 1912 from Yunnan Province, *Rhopalopsoledulongjianga* Yang & Du, sp. nov. Additionally, we found *Rhopalopsolebispina* (Wu, 1949) in this batch of specimens and provided a high-definition picture.

## Materials and methods

Specimens were collected by hand and preserved in 75% ethanol. Morphological details were examined with a Leica MZAPO microscope. Colour illustrations were taken with a KEYENCE VHX-5000. All specimens used in this study are deposited in the Insect Collection of Yangzhou University (ICYZU), Jiangsu Province, China. The morphological terminology follows that of Sivec et al. (2008).

## Taxon treatments

### Rhopalopsole dulongjianga

Yang & Du sp. nov.

B677DF8C-F652-53D0-B581-AC8FF7D22EAA

E2CA530C-A1F9-4D3C-B3F1-C926E57EFC41

#### Materials

- **Type status:** Holotype. **Occurrence:** recordedBy: Yang Xiao and Zeng Liang-Liang; sex: male; lifeStage: adult; occurrenceStatus: present; occurrenceID: 1DC7F786-9DFF-5643-AA72-A7FEE8FDBBD7; **Taxon:** scientificName: *Rhopalopsoledulongjianga*; class: insecta; order: Plecoptera; family: Leuctridae; genus: Rhopalopsole; specificEpithet: *dulongjianga*; **Location:** stateProvince: Yunnan; county: Nujiang Lisu Autonomous Prefecture; locality: Dulongjiang town; minimumElevationInMeters: 1397; maximumElevationInMeters: 1397; decimalLatitude: 27.841533; decimalLongitude: 98.328782; **Event:** year: 2024; month: 6; day: 4; **Record Level:** institutionID: ICYZU

#### Description

**Figure 1.**
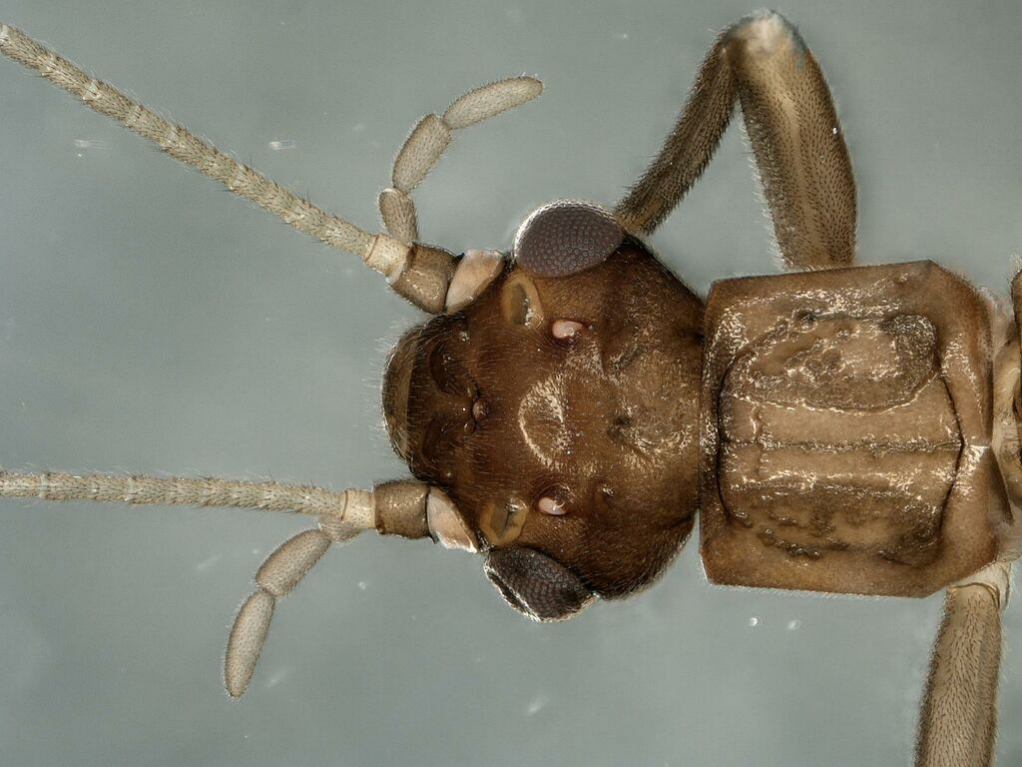
*Rhopalopsoledulongjianga*, sp. nov. Male head and pronotum, dorsal view.

**Figure 2.**
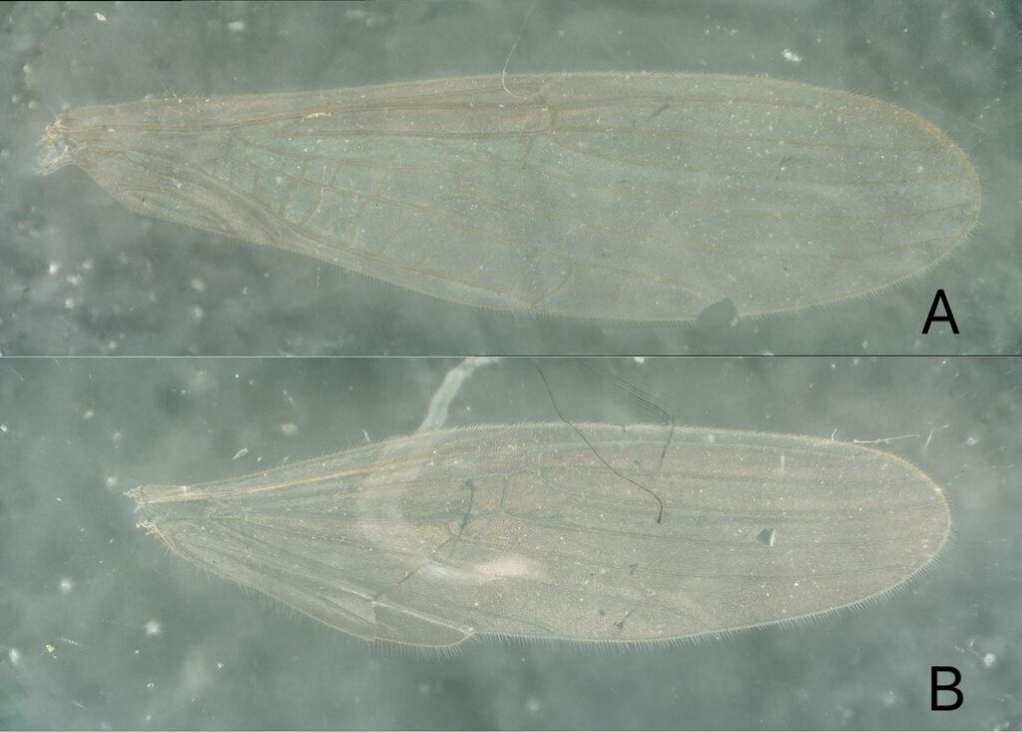
*Rhopalopsoledulongjianga*, sp. nov. **A** Male fore-wings; **B** Male hind-wings.

**Figure 3.**
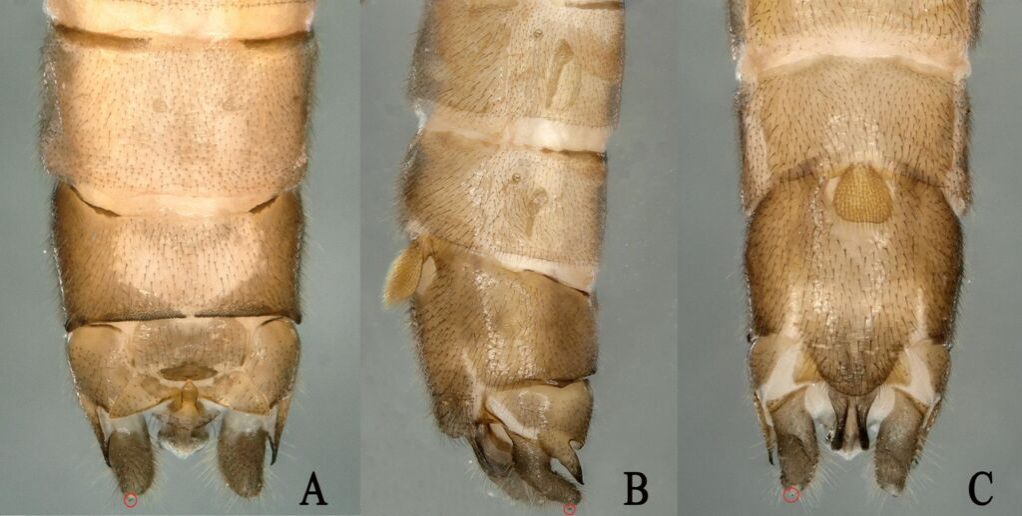
*Rhopalopsoledulongjianga*, sp. nov. **A** Male terminalia, dorsal view; **B** Male terminalia, lateral view; **C** Male terminalia; ventral view.

**Male.** Body length 5.7 mm. Fore-wings length 5.5 mm, hind-wings length 4.2 mm. Head dark brown, wider than pronotum; ocelli pale brown, antennae and palpi light brown. Pronotum brown, quadrate. Legs brown. Wings hyaline and veins light brown (Figs 1, 2). Tergum 9 mostly sclerotised, with the median pentagonal area being somewhat less sclerotised and featuring paired posterior process with sensilla basiconica. Sternum 9 basally with subcircular vesicle bearing dense hairs, apically with a comparatively longer subgenital plate. Tergum 10 bears a large central plate covered with a broad sensilla basiconica patch in the lower half and slightly sclerotised in the upper half. Transverse plates nearly triangular and have some setae. Lateral projections of tergum 10 gradually and regularly taper to a sharp, slightly upturned point. Epiproct thick at base, rod-like, with its tip narrowing and turned forwards. Subanal lobes long and upturned with distinct ventral flaps and longitudinal furrows covering the ventral surface. Cercus hairy and upcurved, with a small spine (Figs 2, 3).

**Female.** Unknown.

**Egg and nymph.** Unknown.

#### Diagnosis

*Rhopalopsoledulongjianga* is similar to menbers of the *R.magnicerca* group (Sivec et al. 2008). The new species closely resembles *Rhopalopsolebispina* (Wu, 1949), with both species sharing similar shapes of the epiproct and the central plate of tergum 10. In *R.dulongjianga*, the cercus is hairy and upcurved, with a small spine and tergum 9 is mostly sclerotised, somewhat less so in the median pentagonal area with a paired posterior process with sensilla basiconica. In contrast, *R.bispina* lacks a spine on the cerci and the hind border of tergum 9 features a band of knob-like ornamentations, narrowly connected in the middle and broadly expanded on each side.

#### Etymology

This new species is named after its collection site, Dulongjiang Town.

#### Distribution

China, Yunnan (Fig. 6).

### Rhopalopsole bispina

(Wu, 1949)

07C5C549-5A8B-5F45-BC03-FB062E7E8A58

#### Materials

- **Type status:** Other material. **Occurrence:** recordedBy: Yang Xiao & Zeng Liang-Liang; sex: 2 males; lifeStage: 2 adult; occurrenceStatus: present; occurrenceID: 93CAE033-74C7-58B7-A4AB-C11B227C172B; **Taxon:** scientificName: *Rhopalopsolebispina*; class: Insecta; order: Plecoptera; family: Leuctridae; genus: Rhopalopsole ; specificEpithet: *bispina*; **Location:** country: China; stateProvince: Yunnan; county: Nujiang Lisu Autonomous Prefecture; locality: Dulongjiang town; minimumElevationInMeters: 1400; decimalLatitude: 27.841534; decimalLongitude: 98.328783; **Event:** year: 2024; month: 6; day: 14; **Record Level:** institutionID: ICYZU

#### Description

**Figure 4.**
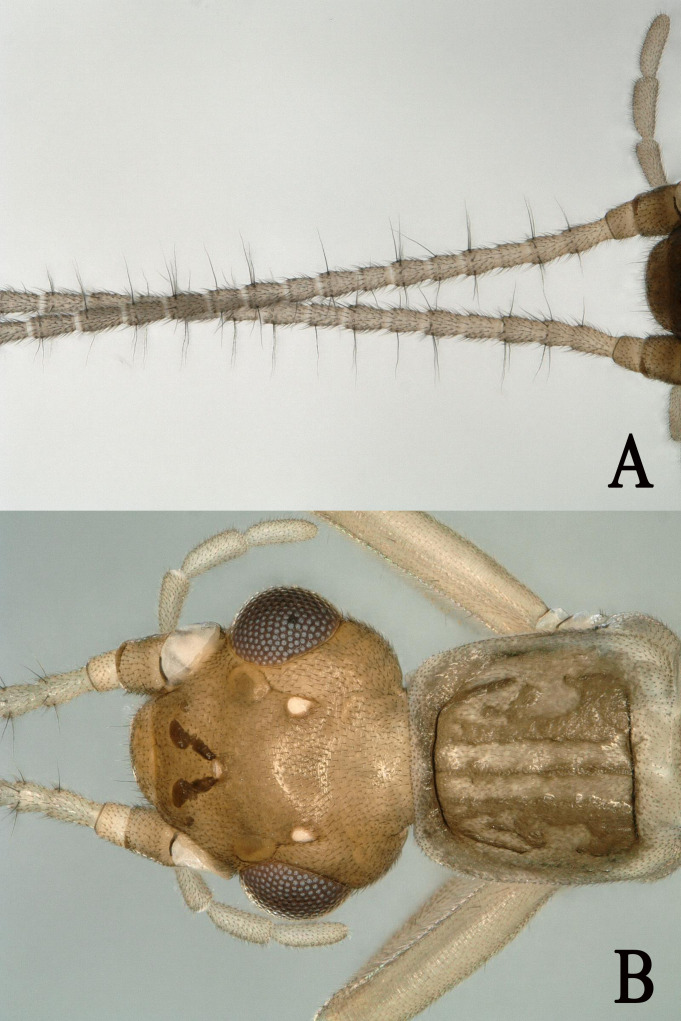
*Rhopalopsolebispina* (Wu, 1949). **A** Male head and pronotum, dorsal view; **B** Male antennae, dorsal view.

**Figure 5.**
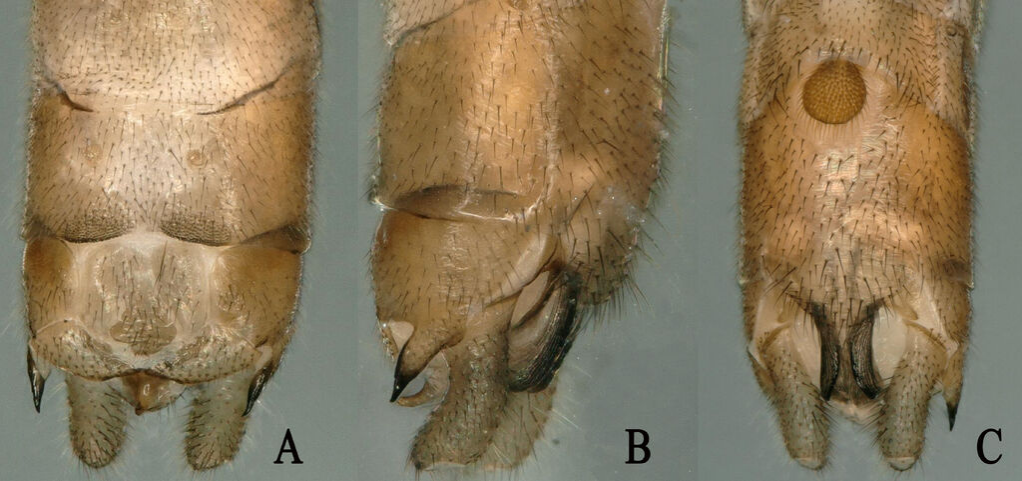
*Rhopalopsolebispina* (Wu, 1949). **A** Male terminalia, dorsal view; **B** Male terminalia, lateral view; **C** Male terminalia, ventral view.

**Figure 6.**
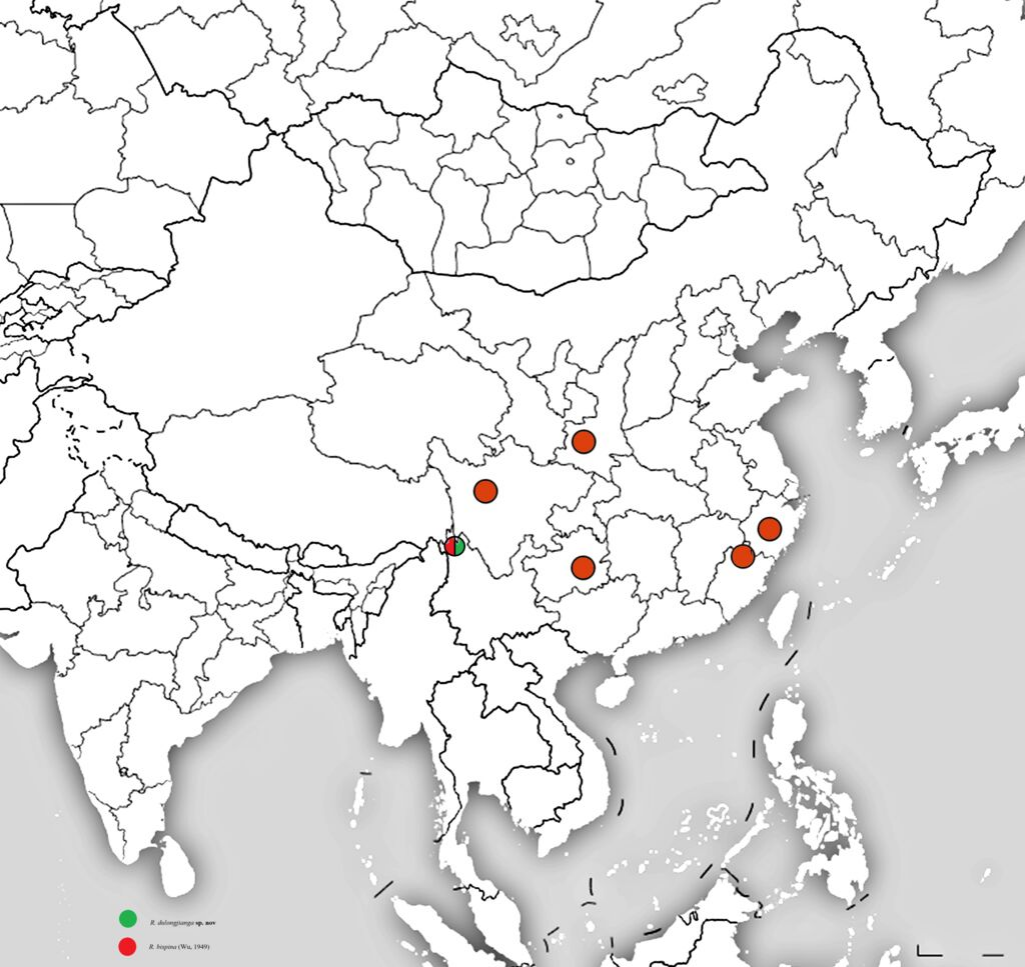
The distribution of *R.dulongjianga* **sp. nov** and *Rhopalopsolebispina* (Wu, 1949) in China.

This species was well re-described by Sivec et al. (2008) (Fig. 4). Additionally, we observed that the antennae of *R.bispina* have long hairs. Therefore, we provide only colour picture in this paper (Figs 4, 5) and a map of the distribution of *R.bispina* in China (Fig. 6).

#### Distribution

China, Guizhou; Sichuan; Zhejiang; Yunnan; Fujian.

## Identification Keys

### Key to the males of *Rhopalopsole* species from Yunnan Province of China

**Table:** 

1	Lateral processes of tergum 10 bifurcate	2
–	Lateral processes of tergum 10 not bifurcate	4
2	Epiproct triangulate in dorsal view	*R.brevidigitata* Qian & Du, 2017
–	Epiproct not triangulated in dorsal view	3
3	Antennae with long hairs	*R.sinensis* Yang & Yang, 1993
–	Antennae without long hairs	*R.yunnana* Sivec & Harper, 2008
4	Subanal lobes are divided into three parts	*R.dentiloba* (Wu, 1973)
–	Subanal lobes are not divided into three parts	5
5	Lateral projections of tergum 10 nearly parallel-sided in lateral view	*R.emeishan* Sivec & Harper, 2008
–	Lateral projections of tergum 10 gradually taper towards the apex in the lateral view	6
6	Tergum 10 with central sclerite is about the same length and width	*R.faciursina* Qian & Du, 2017
–	Tergum 10 with central sclerite distinctly broader than long	7
7	Tergum 9 with a T-shaped weakly sclerotised area in the median	*R.siculiformis* Qian & Du, 2012
–	Tergum 9 without a T-shaped weakly sclerotised area in the median	8
8	The cercus with a small spine and tergum 9 is mostly sclerotised, somewhat less so in the median pentagonal area with a paired posterior process with sensilla basiconica	*R.dulongjianga* Yang & Du, sp. nov.
–	The cercus without spine and tergum 9 without a pentagonal weakly sclerotised area in the median	*R.bispina* (Wu, 1949)

## Supplementary Material

XML Treatment for Rhopalopsole dulongjianga

XML Treatment for Rhopalopsole bispina
